# Metabolic effects of a late hypotensive insult combined with reduced intracranial compliance following traumatic brain injury in the rat

**DOI:** 10.3109/03009734.2010.503906

**Published:** 2010-10-27

**Authors:** Konstantin Salci, Per Enblad, Michel Goiny, Charles F. Contant, Ian Piper, Pelle Nilsson

**Affiliations:** ^1^Department of Neurosurgery, Uppsala University Hospital, UppsalaSweden; ^2^Department of Physiology and Pharmacology, Karolinska Institute, StockholmSweden; ^3^Department of Neurosurgery, Baylor College of Medicine, HoustonUSA; ^4^Institute of Neurological Sciences, Southern General Hospital, GlasgowUK

**Keywords:** Hypotension, intracranial compliance, microdialysis, rat, traumatic brain injury

## Abstract

**Introduction:**

Traumatic brain injury makes the brain vulnerable to secondary insults. Post-traumatic alterations in intracranial dynamics, such as reduced intracranial compliance (IC), are thought to further potentiate the effects of secondary insults. Reduced IC combined with intracranial volume insults leads to metabolic disturbances in a rat model. The aim of the present study was to discern whether a post-traumatic hypotensive insult in combination with reduced IC caused more pronounced secondary metabolic disturbances in the injured rat brain.

**Materials and methods:**

Rats were randomly assigned to four groups (*n* = 8/group): 1) trauma with hypotension; 2) trauma and reduced IC with hypotension; 3) sham injury with hypotension; and 4) sham injury and reduced IC with hypotension. A weight drop model of cortical contusion trauma was used. IC was reduced by gluing rubber film layers on the inside of bilateral bone flaps before replacement. Microdialysis probes were placed in the perimeter of the trauma zone. Hypotension was induced 2 h after trauma. Extracellular (EC) levels of lactate, pyruvate, hypoxanthine, and glycerol were analyzed.

**Results:**

The trauma resulted in a significant increase in EC dialysate levels of lactate, lactate/pyruvate ratio, hypoxanthine, and glycerol. A slight secondary increase in lactate was noted for all groups but group 2 during hypotension, otherwise no late effects were seen. There were no effects of reduced IC.

**Discussion:**

In conclusion, reduced IC did not increase the metabolic disturbances caused by the post-traumatic hypotensive insult. The results suggest that a mild to moderate hypotensive insult after initial post-traumatic resuscitation may be tolerated better than an early insult before resuscitation.

## Introduction

Traumatic brain injury (TBI) is a major cause of death and disability globally (1,2). Outcome for patients with TBI has improved over the past decades due to improved pre-hospital and neurointensive care that has focused on avoiding/reducing secondary insults in these patients (3–8) rather than through use of new pharmacological treatments for TBI. Despite promising results from experimental drug trials, none of the clinical trials of new drugs have been able to show a significant effect on outcome for TBI patients (2). It appears that further improvements in care require continued focus on pathophysiological mechanisms responsible for the enhanced vulnerability of the brain to secondary insults after trauma. Understanding the effects of different secondary insults requires multimodality monitoring to elucidate each insult's effects on the tissue and at what time point the different insults are dangerous to the patient.

Systemic hypotension is an insult that occurs in 15%–35% of patients with severe head injury and is associated with a significant increase in mortality and morbidity (9–16). Both early (from injury through resuscitation) and late (in the intensive care unit (ICU)) post-traumatic systemic hypotension aggravates TBI (14,16). Transient hypotension is common in the ICU in TBI patients and can be associated with poorer outcome (17). A recent clinical study has shown that post-traumatic hypotension independently increased the risk of mortality, but it did not increase mortality in TBI patients more than it did for non-TBI patients (18).

Experimental studies have also shown synergistic effects of TBI and early secondary systemic hypotension resulting in increased contusion volume (19) and reduction of high-energy phosphates (ATP) (20), cerebral blood flow (CBF) (21), EEG activity (21), and cerebral oxygen delivery (22). In one TBI model, post-traumatic hypotension and hypoxia had different degrees of impact on brain energy metabolism depending on the timing of when the insult occurred (23,24).

The status of intracranial pressure/volume dynamics is another factor that influences the effect of secondary insults upon TBI. Reduced intracranial compliance (IC), i.e. reduced ability to compensate for additional intracranial volume, is a common clinical situation following a traumatic brain injury. A reduction of IC may under certain circumstances lead to hemodynamic effects followed by metabolic disturbances and ischemia (25–28). It seems that reduced IC after trauma may enhance the effect of various secondary insults and is an important observation with implications for the management of head-injured patients. In order to facilitate basic studies of these mechanisms a rodent model of TBI has been established where it is possible to decrease IC/pressure volume index (PVI) with negligible effects on intracranial pressure (ICP) (29). Results from this model show that decreased IC, in combination with superimposed ICP insults, can lead to metabolic disturbances in the tissue (30).

Many experimental studies focus on the direct brain tissue effects of TBI in isolation. This pathophysiology is important to study, but it is very difficult to intervene clinically at this phase because of the inevitable delays between the primary insult and initiation of emergency care. Experimental models resembling the post-resuscitation phase in treatment are important to study to increase our understanding of the optimal timing and treatment approach to the patient. The aim of the present experimental study was to determine whether a late post-traumatic hypotensive insult caused a secondary metabolic disturbance in the rat brain following focal cortical injury and whether reduced IC had an impact on such a response.

## Materials and methods

Thirty-two male Sprague-Dawley rats (360–540 g) (ALAB, Stockholm, Sweden) were used and had free access to food pellets and water. Anesthesia was induced by placing the rats in a gas mixture of Halothane^®^ 3% and O_2_:N_2_O (1:1). Thereafter, they were intubated and mechanically ventilated (Ugo Basil Rodent Ventilator, Varese, Italy). Anesthesia was maintained with isoflurane (1.2%–1.8%) and O_2_:N_2_O (1:2). Arterial and venous catheters were surgically implanted into tail vessels and the right femoral artery. Mean arterial blood pressure (MABP) was continually measured. Arterial blood gases were checked regularly throughout the experiment and were maintained within normal levels, i.e. pCO_2_ 4.5–5.5 kPa, pO_2_ 12–20 kPa, and pH 7.35–7.45. Body temperature was monitored with a rectal probe and kept between 37.0–37.5°C with a heating pad. After catheter preparation the animals were placed in a stereotaxic frame. The skull was exposed and a burr hole (1.5 mm diameter) was made 1 mm caudal to bregma and 1.6 mm lateral to the mid-line for access to the left lateral ventricle. Bilateral craniotomies (6 × 9 mm) were made over the parietal cortex with the center 3 mm caudal to the bregma on the right side and slightly more caudal on the left side due to the ventricular burr hole (29).

A two-way single lumen system (PK Kit IDT-XX, Ohmeda Pte Ltd, Singapore), filled with physiological saline, was used for intracranial pressure (ICP) measurements. The tubing system was in open communication between the left lateral ventricle and a pressure transducer. An amplifier (Transducer Interface, Harvard Apparatus, Edenbridge, England) was connected to a computer running LabWindows CVI software for continuous online acquisition, display, and storage of the ICP signal. A 24-gauge spinal needle was used as a ventricular catheter (outer diameter 0.55 mm). It was stereotactically inserted in the left ventricle. During insertion continuous registration of ICP was carried out. Intraventricular placement was confirmed by the sudden decrease in ICP upon ventricular puncture, a rapid increase in ICP due to jugular vein compression, and the presence of cardiopulmonary pulsation on the monitor screen. The ventricle was punctured at a depth of 3.0–3.5 mm from the dura. For ICP recording the zero point was adjusted to the level of the external auditory canals. ICP was recorded continuously.

A microdialysis (MD) probe was stereotactically inserted through a small incision in the dura medially in the perimeter of the trauma zone (right side). The MD probe was perfused for 2 h before the trauma, and the last three 10-minute MD dialysate samples were averaged to obtain a base-line value. Before trauma or sham injury the MD probe and ICP needle were removed and stereotactically reinserted within 1 and 3 min, respectively. The MD probe had a membrane length of 2 mm (MAB 6.14.2; Metalant AB, Stockholm, Sweden). Mock CSF (containing Na^+^ 140 mM, K^+^ 2.7 mM, Ca²^+^ 1.2 mM, Mg²^+^ 0.9 mM, and Cl¯ 147 mM) was perfused through the probe at a flow rate of 2 μL/min using a microinjection pump (CMA/100; CMA/Microdialysis, Stockholm, Sweden). After 1.5 h of equilibration the MD samples were collected in 10-min fractions.

Trauma was produced by dropping a 21 g weight from 35 cm onto a piston (diameter 4.5 mm) resting on the dura. The piston was constructed to allow a maximum compression of 1.5 mm (29,31). Intracranial volume was altered in the different groups by gluing either 0 or 3 layers of rubber film (0.18 mm thick; Kofferdamm medium, Dental AB, Stockholm, Sweden) on the inside of the bone flaps before they were glued back in place. Histoacryl (B Braun Melsungen, Germany) tissue glue was used for both procedures (29). One layer of rubber film constituted a volume of approximately 10 μL.

**Figure 1.**
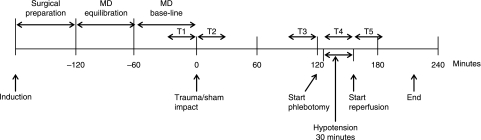
Flow chart of the experiment. Time periods; T1 pre-impact, T2 post-impact, T3 pre-insult, T4 insult, and T5 reperfusion. MD = microdialysis.

The bone flaps were replaced under microscopic control within 5–10 min of impact. Two hours after impact hypotension was induced by withdrawal of blood through the femoral artery. Earlier experiments from our department, using the same degree of TBI, showed that the observed metabolic disturbances after TBI normalized approximately 2 h after impact (31). Heparinized syringes were used to draw blood until the MABP reached approximately 50 mmHg and was maintained at this level for 30 min by further withdrawal of blood or re-injection of blood as needed. Normally 5–8 mL blood withdrawal was required to obtain MABP of 50 mmHg. The blood was kept warm (37°C). After 30 min of hypotension, reperfusion started with slow re-injection of blood as required to obtain normal MABP. See Figure 1 depicting the flow chart of the experimental protocol.

At the end of the experiment, the thorax of the animal was opened and the heart catheterized through the left ventricle wall. The right atrium was opened as an outlet. The animal was perfusion-fixated by infusion of 250 mL of physiological saline followed by 250 mL of 4% formaldehyde solution. The animal was decapitated and the brain kept in 4% formaldehyde solution. The brains were cut in coronal sections over the sites of the ventricular needle, MD probe, and craniotomy areas, and analyzed under the operation microscope.

Analyses of the MD fractions (random samples) were performed. Analyses of energy metabolites (lactate, pyruvate, and hypoxanthine) were made in order to assess the metabolic state. Glycerol was analyzed as a marker of cell membrane degradation. Lactate, pyruvate, and glycerol were analyzed with an enzymatic colorimetric method using the CMA/600 microdialysate analyzer (CMA Microdialysis, Stockholm, Sweden). Hypoxanthine was measured using high-performance liquid chromatography with UV detection at 254 nm. Briefly, a reverse phase 100 × 4 mm nucleosil-100 C18 column (Knauer GmbH, Germany) was eluted with a mobile phase constituted of 0.01 M sodium phosphate (pH 6) and 6% methanol. The signal of the UV detector (BAS, USA) was evaluated with an electronic SP4290 integrator (Spectra Physics, USA) against freshly prepared standard solutions.

### Study design and statistical methods

The animals were randomly assigned to one of the following four groups (*n* = 8/group): Sham injury with 0 or 3 layers of rubber film and hypotensive insult (groups Sh0 and Sh3), or trauma injury with 0 or 3 layers of rubber film and hypotension insult (groups Tr0 and Tr3). For statistical analyses the experiment was divided into five time periods: T1: 30 min before trauma (base-line or pre-impact period), T2: 30 min after trauma (post-impact period), T3: 30 minutes before hypotension (pre-insult period), T4: over the 30 min hypotensive insult (insult period), and T5: 30 min after the insult (reperfusion period) (Figure 1).

For each of the variables the average per animal was calculated for each time period. One animal (Sh0) had missing data for the blood pressure and ICP at T5 due to sudden death during this period. Hypoxanthine values were missing for two animals in group Tr0 (one at all time periods and the other at T3, T4, and T5) due to technical problems. Some animals had extra MD measurements due to the extra time needed from the start of phlebotomy until BP reached near to 50 mmHg, and these extra measurements were deleted from all analyses.

The averages for each animal and time period were then analyzed in an ANCOVA model including the factors: treatment-group (the four groups Sh0, Sh3, Tr0, and Tr3), time, and the interaction term (treatment-group*time). ICP was included as a covariate in the analyses of all variables except ICP. The statistical significance of each effect was determined from the model, and the pair-wise comparisons between groups and time periods were also derived from the model. A probability of 0.05 or less was considered as statistically significant.

As the residuals for the variables L/P ratio (lactate/pyruvate ratio), glycerol, hypoxanthine, and lactate were extremely skewed, these variables were transformed to their natural logarithms before they were analyzed. The data were also summarized by means, standard deviations, medians, minimum, and maximum values per treatment group and time.

### Ethics

The experimental protocol was approved by the ethics committee for Uppsala University.

## Results

### Physiological parameters and macromorphology

Blood gases, temperature, and BP for each group prior to impact are shown in Table I. Blood gases and temperature were maintained at these levels throughout the experiment, with the exception that blood gases could not be checked during the hypotensive insult or reperfusion periods, due to practical reasons. There were no signs of hematoma on the coronal sections of the perfused brains.

**Table I.** The different groups and physiological parameters prior to impact.

Group	*n*	Injury	Rubber film layers	Hypotension	PO_2_ (kPa)	PCO_2_ (kPa)	pH	Temp (°C)	MABP (mmHg)
Sh0	8	Sham	0	Yes	17.9 ± 2.3	5.33 ± 0.31	7.39 ± 0.030	37.1 ± 0.14	90.8 ± 5.92
Sh3	8	Sham	3	Yes	19.0 ± 1.7	5.01 ± 0.35	7.39 ± 0.036	37.0 ± 0.14	91.7 ± 10.3
Tr0	8	Trauma	0	Yes	18.4 ± 1.9	5.03 ± 0.75	7.39 ± 0.044	37.1 ± 0.14	86.3 ± 7.74
Tr3	8	Trauma	3	Yes	17.6 ± 1.9	5.05 ± 0.36	7.38 ± 0.023	37.0 ± 0.13	89.4 ± 6.80

Values are shown as mean ± SD. Group definition: Sh/Tr: sham or trauma injury (0/3 = no. of layers).

### Overall effects

**Figure 2.**
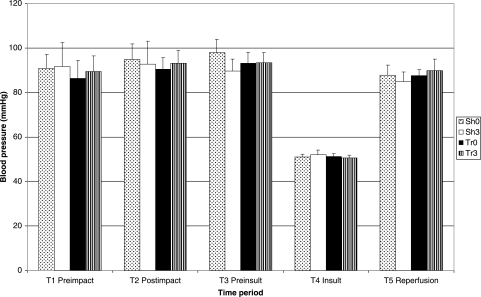
Blood pressure during the time periods (mean ± SD of 30 time points/time period). For statistics and significance see Tables II and III. Time periods T1–T5. Sh0/3 and Tr0/3 = sham/trauma injury with 0/3 layers of rubber film.

**Figure 3.**
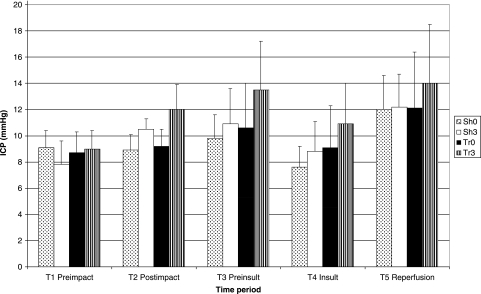
ICP during the time periods (mean ± SD of 30 time points/time period). For statistics and significance see Tables II and III. Time periods T1–T5. Sh0/3 and Tr0/3 = sham/trauma injury with 0/3 layers of rubber film.

**Figure 4.**
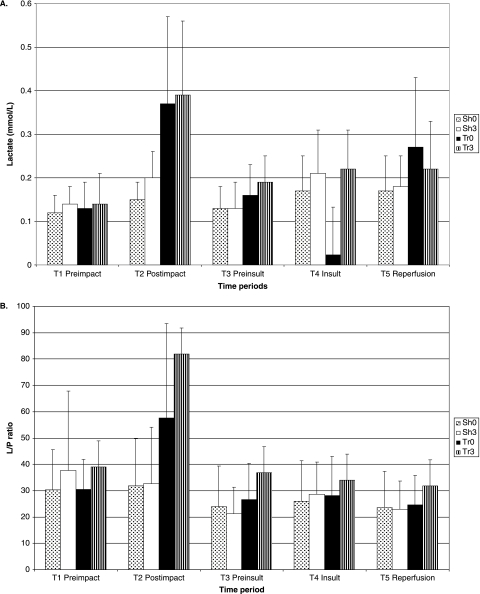

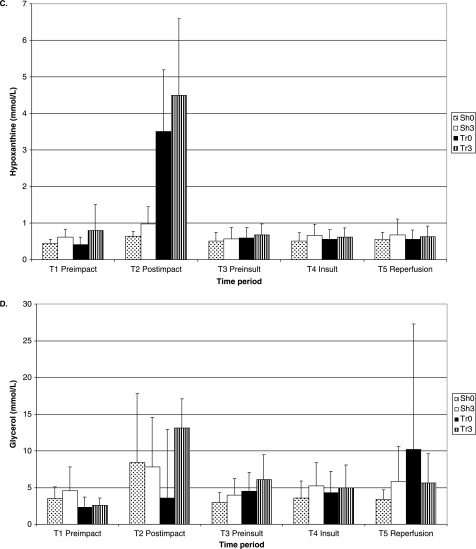
Lactate (A), L/P ratio (B), hypoxanthine (C), and glycerol (D) during the time periods (mean ± SD of 3 samples/time period). For statistics and significance see Tables II and III. T1–T5: time periods. Sh0/3 and Tr0/3 = sham/trauma injury with 0/3 layers of rubber film.

Figures 2–4 and Table II contain the overall results of the analyses for BP, ICP, and each of the dialysates. The time effect and the interaction between group and the time of measurement were the most commonly significant results. A significant time effect indicates that the means at each time differed from each other. The time-by-group interaction indicates that the difference in the mean between the different groups depended on time. For ICP this was mainly due to a layers–time interaction and for the dialysates mainly due to an injury–time interaction. A more detailed presentation of the results follows below.

**Table II.** Probability values for the different effects.

Dependent	Group	Time	ICP	Group*Time
1. Blood Pressure	0.4821	<0.0001	0.0929	0.0689
2. ICP	0.1577	<0.0001	–	0.0464
3. Log L/P ratio	0.2774	<0.0001	0.1229	0.0007
4. Log Glycerol	0.6700	<0.0001	0.8954	0.0108
5. Log Hypoxanthine	0.0971	<0.0001	0.0418	<0.0001
6. Log Lactate	0.1871	<0.0001	0.4357	<0.0001

Table III shows *P* values for comparisons within and between groups for different time periods (all time periods not shown).

**Table III.** Comparisons within and between groups (*P* values from contrasts in ANCOVA model).

	Blood Pressure	ICP	Lactate	L/P ratio	Hypoxanthine	Glycerol
T2–T1, Sh0	0.0788	0.7773	0.0807	0.9197	0.0152	0.0555
T2–T1, Sh3	0.3047	0.0004	0.0036	0.4605	0.0007	0.0525
T2–T1, Tr0	0.0489	0.4717	<0.0001	0.0001	<0.0001	<0.0001
T2–T1, Tr3	0.0315	0.0001	<0.0001	<0.0001	<0.0001	<0.0001
T2–T1, Sh3–Sh0	0.6418	0.0060	0.3438	0.5463	0.3964	0.9238
T2–T1, Tr0–Sh0	0.8761	0.4785	<0.0001	0.0065	<0.0001	0.0005
T2–T1, Tr3–Sh3	0.3818	0.7826	<0.0001	0.0001	<0.0001	0.0009
T2–T1, Tr3–Tr0	0.8159	0.0198	0.6115	0.3985	0.4634	0.9520
T4–T3, Sh0	<0.0001	0.0034	0.0423	0.2167	0.6306	0.6375
T4–T3, Sh3	<0.0001	0.0055	0.0010	0.0145	0.5647	0.3480
T4–T3, Tr0	<0.0001	0.0487	0.0092	0.5055	0.6444	0.6548
T4–T3, Tr3	<0.0001	0.0006	0.5051	0.9506	0.1772	0.4177
T4–T3, Sh3–Sh0	0.0040	0.9094	0.3348	0.3668	0.4366	0.7324
T4–T3, Tr0–Sh0	0.0888	0.4823	0.6926	0.6669	0.9819	0.5052
T4–T3, Tr3–Sh3	0.1017	0.6286	0.0504	0.0650	0.1522	0.1980
T4–T3, Tr3–Tr0	0.6699	0.2843	0.1640	0.6011	0.5595	0.7783

T1–T4 = time periods; Sh/Tr = sham or trauma injury (0 or 3 indicates number of layers).

### Trauma effect and the influence of layers

The changes in BP are illustrated in Figure 2 and Table III. The average BP before impact in the different groups varied between 86 mmHg and 92 mmHg. The impact resulted in a slight (4.5–5 mmHg) but significant BP increase in the trauma injury groups (Tr0 and Tr3). Figure 3 shows ICP by time. Base-line (pre-impact) ICP was 7.75–9.08 mmHg. During the post-impact period there was a slight but significant ICP increase in the groups with three layers (Table III). In the groups with zero layers there was no statistically significant difference. The injury itself did not increase ICP during the post-impact period. During the pre-insult period there was no statistically significant difference in ICP between the groups.

Figure 4 shows neurochemical changes over time. The average base-line values were: lactate 0.12–0.14 mmol/L; L/P ratio 30–39; hypoxanthine 0.41–0.79 micromol/L; glycerol 2.32–4.58 micromol/L. The trauma resulted in a multifold increase for all metabolites during the post-impact period in the trauma injury groups, whereas in the sham injury groups a slight increase was seen that reached significance for hypoxanthine (groups Sh0 and Sh3) and lactate (group Sh3) (Table III). The increase during the post-impact period was significantly higher for the trauma injury groups as compared to the sham injury groups. There was no effect of reduced compliance during the post-impact period.

### Hypotensive insult effect and influence of layers

The hypotensive insult resulted in a decrease in BP for all groups (Figure 2, Tables II and III). During the hypotensive insult there was a consistent decrease in ICP for all groups compared to the pre-insult period (Figure 3 and Table III). The reperfusion resulted in the subsequent return to or slight overshot of pre-insult ICP levels. The effect of hypotension on ICP was the same for all groups.

The neurochemical changes induced by the hypotensive insult are shown in Figure 4. The insult caused a slight but statistically significant increase in L/P ratio for group Sh3 and a slight increase in lactate compared to pre-insult values for all groups, reaching statistical significance for all except Tr3. During the reperfusion period the L/P ratio decreased for group Sh3 to pre-insult values. Lactate remained increased during reperfusion in the trauma injury groups. During the reperfusion period lactate remained higher than the pre-insult period for all groups except Tr3. For the other dialysates no significant effects were seen. There were no obvious effects related to layers (e.g. compliance level) during the insult or reperfusion periods.

## Discussion

The present study shows that a late hypotensive insult, following a focal traumatic brain injury, affects brain energy metabolism with increases in lactate and L/P ratio but not to the extent that the energy depletion leads to AMP or membrane degradation. Reduced IC does not lead to a more pronounced disturbance. The results from this study are similar to the metabolic and membrane responses seen after diffuse axonal injury combined with late hypotension and hypoxia (24). These results suggest that both diffuse and focal brain injury have similar temporal patterns for tissue reaction in the acute phase after the trauma. The mild metabolic disturbance from a delayed hypotensive insult compared to the more pronounced changes seen in an early hypotensive insult helps to illustrate the complex dynamics of intracranial pathophysiology in TBI where very short time spans can lead to changes in tissue reactions to a secondary insult.

Brain tissue survival in traumatic injury is dependent on adequate supplies of oxygen and glucose so that ATP production can be maintained, as is the case in all central nervous system injury. The difference in tissue response between early and late insults is probably related to effects of relative ischemia. Earlier studies in a TBI model similar to the present one, but without reduced intracranial volume, have shown: increases of extracellular excitory amino acid levels (31), increase of interstitial [K^+^] and decrease of interstitial [Ca^2+^] (32), and post-traumatic seizures immediately following the trauma (33). All of these disturbances result in an increased energy demand. In this model it has also been shown that there is a decrease in cerebral blood flow directly after the trauma that persists for 80 minutes (34). The combined effects of these disturbances lead to metabolic disturbances that normalize by 120 minutes *post* trauma (31).

In the present model, which is a modification of the original model (31), it has also been shown that metabolic disturbances normalize within 120 minutes (30). During this recovery period the tissue is sensitive to secondary insults. If the injured brain is subjected to ICP insults a more profound metabolic disturbance occurs with increases in interstitial levels of hypoxanthine and glycerol in the trauma group with reduced IC (30). The mild disturbances in metabolism seen in the present study show that once the tissue has recovered from the trauma then it can tolerate at least one secondary insult at a time even if IC is reduced. Another factor that may influence the present response is that a hypotensive insult of this magnitude will lead to a decrease in cerebral blood volume, which in turn lowers ICP.

At what level physiological changes become insults is a question that is central in discussions concerning intensive care. That the hypotensive insult in this study is too mild to cause metabolic disturbances in the tissue is not supported by findings from other studies using the same level of hypotension. Geeraerts et al. (24) have shown that TBI and a combined secondary insult of hypotension and hypoxia leads to metabolic disturbances. In their study, with two different time points for a 15-minute long combined insult of hypoxia (PaO_2_ 5.3 kPa) and hypotension (MABP 40 mmHg), there was an increase of lactate and L/P ratio at both time points. The late insult at 225 minutes *post* trauma did not affect cerebral metabolism to the same extent, while the early insult at 25 minutes had a more pronounced effect. These studies also showed that the hypotensive insult itself led to metabolic disturbances if it occurred at 25 minutes *post* sham trauma and that this disturbance was of the same magnitude as that seen with a late insult after trauma. All of these responses show that the insult is sufficient to elicit a metabolic reaction in the tissue.

The findings from the present study, earlier studies with this model, and the studies by Geeraerts et al. in a different model, but with similar results, shed light on the problems related to development of neurocritical care in TBI. Earlier, experimental studies were a way of dealing with the complex pathology related to TBI. Most studies have focused on a single or a few phenomena during the acute phase and have used continuous or snap-shot methods to monitor changes. Because of practical and economical reasons most experimental studies only cover a few hours of observation. In contrast, clinical studies have had access to continuous monitoring, around the clock and every day of the week, for as long as the patient has been in the ICU. However, these studies have relied on paper documentation of physiological parameters which has only given summary information on care and pathophysiological disturbances. With the advent of modern computers it is now possible to achieve detailed documentation of the pathophysiology occurring with the patient over longer periods of time (35). To fully understand the impact of the type of insult studied in this paper one must move on into the clinical setting and use long-term, multimodality, high-resolution monitoring in the resuscitated patients. With this type of approach it will be possible to identify which findings from the laboratory environment can be applied in the clinical setting.
